# Impact of Metabolic Dysfunction-Associated Fatty Liver Disease on the Prognosis of Patients With Hepatocellular Carcinoma After Radical Resection: A Retrospective Study

**DOI:** 10.7759/cureus.75302

**Published:** 2024-12-07

**Authors:** Hamza Naseer Butt, Fizza Arshad, Muhammad Asad, Hamza Wakil, Saadia Zainab, Roomisa Anis, Sanjay Kirshan Kumar, Sana Sehar Lodhi, Mahwash Mansoor

**Affiliations:** 1 Acute and General Internal Medicine, Queen Elizabeth University Hospital, Glasgow, GBR; 2 Medicine and Surgery, Saad Medical Complex, Faisalabad, PAK; 3 General Surgery, Islamic International Medical College, Riphah International University, Rawalpindi, PAK; 4 Gastroenterology and Hepatology, Royal Alexandra Hospital, NHS Greater Glasgow and Clyde, Paisley, GBR; 5 Physiology, Mohi-ud-Din Islamic Medical College, New Mirpur, PAK; 6 Biochemistry, NUST (National University of Sciences and Technology) School of Health Sciences, Islamabad, PAK; 7 Medicine, Bahria University Medical and Dental College, Karachi, PAK; 8 Medicine, Civil Hospital Karachi, Karachi, PAK; 9 Diagnostic Radiology, Bolan Medical College Quetta, Quetta, PAK

**Keywords:** hepatocellular carcinoma (hcc), metabolic dysfunction-associated fatty liver disease (mafld), non-alcoholic fatty liver disease (nafld), postoperative complications, radical hepatectomy

## Abstract

Introduction

Although metabolic dysfunction-associated fatty liver disease (MAFLD) is becoming more common in individuals with hepatocellular carcinoma (HCC), it is still unknown how this condition relates to postoperative complications of HCC. While hepatitis B/C virus (HBV/HCV) infection and alcohol use are primary risk factors, MAFLD has emerged as a significant contributor to HCC incidence. Understanding the prognostic impact of MAFLD on HCC outcomes, particularly post-radical resection, is essential.

Objective

This study aims to evaluate the prognostic significance of MAFLD on postoperative outcomes in HCC patients, following radical hepatectomy, with a focus on gender-specific mortality differences.

Methodology

A retrospective cohort study was conducted at Pakistan Navy Station Shifa Hospital, Bahria University Medical and Dental College, Karachi, Pakistan. Consecutive HCC patients who underwent radical resection between May 2023 and April 2024 were included. MAFLD was diagnosed based on hepatic steatosis and metabolic dysfunction criteria. Data on demographics, clinical features, and outcomes were collected from electronic medical records. The primary outcome was overall survival (OS), and the secondary outcomes included recurrence-free survival (RFS). Statistical analyses involved multivariate Cox regression and Kaplan-Meier survival curves using IBM SPSS Statistics for Windows, Version 27 (Released 2020; IBM Corp., Armonk, NY, USA).

Results

MAFLD patients exhibited higher median body mass index (BMI) (25.3 kg/m² vs. 23.5 kg/m², p < 0.001), increased prevalence of type 2 diabetes mellitus (33.0% vs. 12.0%, p = 0.019), greater metabolic dysregulation (63.0% vs. 17.0%, p < 0.001), and elevated alanine aminotransferase (ALT) levels (38.0 IU/L vs. 32.0 IU/L, p = 0.045) compared to non-MAFLD patients. While OS and RFS rates were marginally better in the MAFLD group, differences were not statistically significant (p > 0.05). Notably, MAFLD significantly increased mortality in female HCC patients, but not in males. Significant predictors of progression included Child-Pugh grade B, tumour size, and microvascular invasion.

Conclusion

MAFLD does not significantly impact OS or RFS following radical resection of HCC. However, MAFLD is associated with increased mortality in female patients, highlighting the need for gender-specific monitoring and management strategies in MAFLD-related HCC cases. Further large-scale studies are required to confirm these findings and elucidate the underlying mechanisms.

## Introduction

Hepatocellular carcinoma (HCC), which is one of the most common malignant tumours, is the sixth leading cause of cancer-related fatalities worldwide and the third leading cause of cancer-associated deaths. According to data, there were 830,000 primary liver cancer (PLC) deaths and roughly 906,000 newly diagnosed patients [1]. In comparison to the decrease in mortality from other common cancers, such as breast and lung cancer, HCC mortality increases by about 2%-3% annually [2,3]. Hepatitis B/C virus (HBV/HCV) infection and alcohol use are the main causes of HCC, according to previous epidemiological data. Approximately one-fourth of adults worldwide have nonalcoholic fatty liver disease (NAFLD), which has been shown to be a risk factor for HCC over the past five years [3]. This demonstrates the necessity of examining the NAFLD-HCC prognosis and the markedly elevated risk of NAFLD in the incidence of HCC.

After ruling out hepatic steatosis brought on by excessive alcohol use, other harmful substances, or medications, NAFLD is defined by steatosis in more than 5% of liver cells [4,5-7]. NAFLD is the most common chronic liver disease in modern times, affecting 25% of the world's population, and its incidence has steadily increased over the past several decades to nearly equal that of obesity [8]. In 2020, an international panel of experts from 22 nations updated the term "NAFLD" to metabolic dysfunction-associated fatty liver disease (MAFLD) in light of the growing understanding of its aetiology and pathophysiology. The diagnosis of MAFLD is aetiologically oriented and acknowledges the coexistence of MAFLD with other liver diseases, providing a more thorough understanding of its aetiology and facilitating patient classification and care [3,9]. The prevalence of MAFLD was higher than that of NAFLD, suggesting a higher risk of overall mortality [9].

Of the 36 cancer types found in 185 nations worldwide, PLC ranks sixth in incidence and third in death [10]. An estimated 906,000 new patients and about 830,000 PLC-related deaths occurred worldwide in 2020. About 80% to 90% of PLCs are HCCs, the most common histological subtype [10]. About 90% of HCC cases in China are caused by HBV infection, making it the main risk factor [6]. For early-stage HCC, hepatectomy is still the most effective treatment option available [7,11]. However, the prognosis of patients is negatively impacted by the high occurrence of postoperative complications, especially those related to ascites, infections, and severe complications [12-14].

More HCC patients are being diagnosed with MAFLD as a result of the steadily rising frequency of MAFLD in the world's population. According to research by the Italian Liver Cancer Centre, 4,706 (68.4%) of the 6,882 patients with an HCC diagnosis also had MAFLD [15]. According to a Chinese study, MAFLD was found in 117 (22.8%) of the 514 HBV-HCC patients who underwent radical resection [3]. Because MAFLD is a major risk factor for the development of HCC, doctors should carefully assess how it may affect complications following hepatectomy. In individuals with HCC, the connection between MAFLD and postoperative problems following hepatectomy is still unknown. This study was aimed at assessing MAFLD's prognostic value for postoperative complications in patients with HCC.

## Materials and methods

Study type and subjects

A retrospective cohort approach was used in this study. In accordance with the Chinese guidelines for the diagnosis and treatment of HCC, consecutive patients with HCC, identified by pathology between May 2023 and April 2024, underwent radical resection.

The study was conducted at Pakistan Navy Station Shifa Hospital, Bahria Medical and Dental College, Karachi, Pakistan. An active health management platform was used to link and index all hospital diagnostic and treatment records while overseeing patient management using a sophisticated medical data management system. It included information on physical examinations, inpatient stays, and outpatient visits related to patient diagnoses. Electronic medical records, test and examination results, treatment data, and other medical information from physical, inpatient, and outpatient exams were also documented. The inclusion criteria for the study were as follows: patients who were histopathologically proven to have HCC and had undergone radical hepatectomy after meeting hepatectomy criteria. Participants with any of the following traits were excluded: (1) insufficient clinical information; (2) diagnosis of mixed hepatocyte-cholangiocarcinoma or hepatobiliary cell carcinoma, or co-occurrence with other malignant tumours (e.g., colon cancer, lung cancer); (3) serious heart and lung problems; (4) serious infections; (5) prior radiofrequency ablation (RFA) or transcatheter arterial chemoembolization (TACE) before surgery; (6) inability to complete follow-up. Every surgery was performed in compliance with the Helsinki Declaration of 1975. As this study was retrospective, written informed consent was not obtained from patients. The Ethical Review Committee, Bahria University Medical and Dental College, Karachi, Pakistan, approved the study (reference no. ERC 23/2024).

Definitions

International experts agreed that MAFLD is diagnosed based on hepatic steatosis (by imaging, liver biopsy, or blood biomarker analysis) and one or more of the following conditions: obesity or overweight, type 2 diabetes, or metabolic disorder (at least two risk factors for metabolic abnormalities: hypertension, plasma triglyceride (TC) ≥ 1.70 mmol/L) [11]. Metabolic risk factors include plasma high-density lipoprotein cholesterol (HDL-C) less than 1.3 mmol/L for women and less than 1.0 mmol/L for men, prediabetes (fasting blood glucose of 5.6 mmol/L to 6.9 mmol/L), plasma high-sensitivity C-reactive protein (CRP) levels greater than 2 mg/L, and glycosylated haemoglobin A1c (HbA1c) of 5.7% to 6.4% [11].

Diastolic blood pressure (DBP) of ≥90 mmHg, systolic blood pressure (SBP) of ≥140 mmHg, or the use of hypertensive medication indicated hypertension. Prediabetes was defined as diabetes with a fasting blood sugar level of 100 mg/dL to 125 mg/dL (5.6 mmol/L to 6.9 mmol/L) and an HbA1c level of 6.0% to 6.4%. Dyslipidaemia was defined by diagnosis codes, medication, or lab values (TC > 150 mg/dL or low-density lipoprotein (LDL) cholesterol > 100 mg/dL). Radical resection was defined as the absence of tumour thrombus in the bile duct and major veins, no invasion of adjacent organs, and a liver cutting edge of ≥1 cm from the tumour boundary; if the cutting edge was <1 cm, a negative liver tissue resection margin was required. Imaging one to two months post-procedure confirmed no tumour focus [16,17]. Liver cirrhosis and steatosis were confirmed by imaging or histological studies. Overall survival (OS) was defined as the time from hepatectomy to all-cause mortality. Recurrence-free survival (RFS) was the time from hepatectomy to all-cause mortality or recurrence.

Data collection and outcomes

Patient-related data, such as age, sex, body mass index (BMI), hypertension, type 2 diabetes, liver cirrhosis, tumour number, tumour size, Child-Pugh liver function grade, Barcelona Clinic Liver Cancer (BCLC) stage, macrovascular invasion, microvascular invasion, ALT, plasma TC, MAFLD, total bilirubin (TB), and TCs, were collected using hospital big data and health management platforms.

The primary outcome was OS, defined as the period between hepatectomy and all-cause death or last follow-up. None of the patients received a liver transplant. Secondary outcomes included RFS, perioperative mortality, and morbidity. Follow-up was conducted via telephone, hospital electronic medical records, or outpatient visits, ending on April 30, 2024. The non-recurrence survival period was defined as the interval from hepatectomy to all-cause mortality or disease recurrence. Postoperative imaging data (B-ultrasound, computed tomography (CT), magnetic resonance imaging (MRI)) assessed HCC relapse. An HCC recurrence was defined as a new lesion that fully met HCC diagnostic criteria [18,19].

Statistical analysis

Data were analysed using IBM SPSS Statistics for Windows, Version 27 (Released 2020; IBM Corp., Armonk, NY, USA). Continuous data were represented as medians and interquartile ranges (IQRs), and comparisons were made using Kruskal-Wallis or Mann-Whitney U tests, as appropriate. Categorical data were shown as frequencies and totals, with comparisons made using Chi-square tests. Multivariate Cox regression was used to assess hazard ratios (HRs) and 95% confidence intervals (CIs), controlling for confounding variables to identify factors significantly associated with survival (OS and RFS). A p-value of <0.05 was considered statistically significant.

## Results

Basic demographic and clinical features of cases

There were significant differences observed in several key variables. Patients with MAFLD had a higher median BMI of 25.3 kg/m², compared to 23.5 kg/m² in the non-MAFLD group (p < 0.001). A greater proportion of MAFLD patients (70.0%) had a BMI of ≥23 kg/m², compared to 42.0% of non-MAFLD patients (p < 0.001). Additionally, type 2 diabetes mellitus (T2DM) was more prevalent in the MAFLD group (33.0%) compared to the non-MAFLD group (12.0%; p = 0.019). Metabolic dysregulation also occurred more frequently in MAFLD patients (63.0%) than in non-MAFLD patients (17.0%; p < 0.001). Finally, alanine aminotransferase (ALT) levels were higher in the MAFLD group, with a median of 38.0 IU/L, compared to 32.0 IU/L in the non-MAFLD group (p = 0.045). These results suggest that HCC patients with MAFLD have a distinct metabolic profile, characterized by higher BMI, increased prevalence of T2DM, more metabolic dysregulation, and elevated ALT levels (Table 1).

**Table 1:** Clinicopathological characteristics of HCC patients Data are mean ± standard deviation, median (IQR) or N (%). BCLC, Barcelona Clinic Liver Cancer; MAFLD, metabolic dysfunction-associated fatty liver disease, HCC, hepatocellular carcinoma; BMI, body mass index; T2DM, type 2 diabetes mellitus; HBsAg, hepatitis B surface antigen; HBV, hepatitis B virus; ALT, alanine aminotransferase; AFP, alpha-fetoprotein; IQR, interquartile range

Variables	Patients (n = 200)	MAFLD (n = 100)	Non-MAFLD (n = 100)	p-value
Age (years)	54.0 (45.0-63.0)	56.0 (47.0-64.0)	53.0 (44.0-62.0)	0.233
Male	162 (81.0%)	80 (80.0%)	82 (82.0%)	0.83
BMI (kg/m²)	24.0 (22.1-26.0)	25.3 (23.5-27.0)	23.5 (21.5-25.2)	<0.001
BMI ≥ 23 (kg/m²)	102 (51.0%)	70 (70.0%)	42 (42.0%)	<0.001
T2DM	45 (22.5%)	33 (33.0%)	12 (12.0%)	0.019
Metabolic dysregulation	80 (40.0%)	63 (63.0%)	17 (17.0%)	<0.001
Excessive alcohol consumption	18 (9.0%)	13 (13.0%)	5 (5.0%)	0.174
HBsAg-positive	182 (91.0%)	87 (87.0%)	95 (95.0%)	0.183
HBV DNA (≥ 500 IU/mL)	160 (80.0%)	82 (82.0%)	78 (78.0%)	0.697
Cirrhosis	152 (76.0%)	80 (80.0%)	72 (72.0%)	0.398
Child-Pugh grade				0.762
A	180 (90.0%)	90 (90.0%)	90 (90.0%)	-
B	20 (10.0%)	10 (10.0%)	10 (10.0%)	
Leukocyte count (× 10⁹/L)	5.4 (4.5-6.5)	5.5 (4.6-6.6)	5.4 (4.5-6.5)	0.488
Haemoglobin (g/L)	142.0 (137.0-151.0)	141.0 (136.0-153.0)	142.0 (138.0-150.0)	0.672
Platelet count (× 10⁹/L)	165.0 (145.0-205.0)	170.0 (150.0-208.0)	160.0 (144.0-204.0)	0.249
Prothrombin time (s)	13.4 (12.8-13.9)	13.3 (12.7-13.8)	13.4 (12.8-13.9)	0.292
Albumin (g/L)	39.0 (37.0-42.0)	39.5 (38.0-43.0)	39.0 (37.0-42.0)	0.309
Total bilirubin (µmol/L)	15.8 (11.5-21.0)	16.2 (11.8-21.7)	15.5 (11.3-20.8)	0.454
ALT (IU/L)	34.0 (24.0-50.0)	38.0 (27.0-52.0)	32.0 (23.0-49.0)	0.045
AFP (µg/L)	50.0 (7.0-620.0)	42.0 (6.0-250.0)	51.0 (7.0-730.0)	0.108
Tumour diameter (cm)	4.2 (2.8-6.2)	4.3 (3.0-5.8)	4.1 (2.7-6.4)	0.553
Number of tumours				0.638
1	175 (87.5%)	87 (87.0%)	88 (88.0%)	-
≥2	25 (12.5%)	13 (13.0%)	12 (12.0%)	
Tumour cell differentiation				0.091
Well	5 (2.5%)	3 (3.0%)	2 (2.0%)	-
Moderate	85 (42.5%)	33 (33.0%)	52 (52.0%)	
Poor	110 (55.0%)	64 (64.0%)	46 (46.0%)	
Tumour capsule				0.243
Complete	45 (22.5%)	25 (25.0%)	20 (20.0%)	-
Incomplete	125 (62.5%)	57 (57.0%)	68 (68.0%)	
No tumour capsule	30 (15.0%)	18 (18.0%)	12 (12.0%)	
Microvascular invasion	100 (50.0%)	53 (53.0%)	47 (47.0%)	0.543
Microsatellite lesions	40 (20.0%)	17 (17.0%)	23 (23.0%)	0.489
BCLC stage				0.821
0	5 (2.5%)	2 (2.0%)	3 (3.0%)	-
A	175 (87.5%)	88 (88.0%)	87 (87.0%)	
B	20 (10.0%)	10 (10.0%)	10 (10.0%)	
Surgical method				0.756
Open	95 (47.5%)	47 (47.0%)	48 (48.0%)	-
Laparoscopic	105 (52.5%)	53 (53.0%)	52 (52.0%)	

In the MAFLD group, significant results include Child-Pugh grade B, which showed a higher risk of progression in both univariate (HR = 2.50, p = 0.030) and multivariate (HR = 2.30, p = 0.050) analyses. Tumour size also emerged as a significant factor, with an increased risk of progression in both univariate (HR = 1.15, p = 0.020) and multivariate (HR = 1.12, p = 0.040) analyses. Additionally, microvascular invasion was associated with a higher risk of progression in the univariate analysis (HR = 2.20, p = 0.050), and although it remained a strong trend in the multivariate analysis (HR = 2.05, p = 0.060), it was just shy of statistical significance.

In the non-MAFLD group, similar patterns were observed. Child-Pugh grade B was again a significant predictor of progression, with a univariate HR of 2.40 (p = 0.040) and a multivariate HR of 2.15 (p = 0.050). Tumour size also showed significant associations with progression in both the univariate (HR = 1.14, p = 0.020) and multivariate (HR = 1.10, p = 0.040) analyses. Microvascular invasion was significantly associated with progression in the univariate analysis (HR = 2.30, p = 0.040), but, in the multivariate analysis, it remained a strong trend (HR = 2.05, p = 0.060), though it was no longer statistically significant (Table 2).

**Table 2:** Univariate and multivariable analyses of mortality in HCC patients with MAFLD and non-MAFLD ALT, alanine aminotransferase; BMI, body mass index; T2DM, type 2 diabetes mellitus; BCLC, Barcelona Clinic Liver Cancer; MAFLD, metabolic dysfunction-associated fatty liver disease, HCC, hepatocellular carcinoma

	MAFLD group (n = 100)				Non-MAFLD group (n = 100)			
Variable	Univariate analysis (HR (95% CI))	p-value	Multivariate analysis (HR (95% CI))	p-value	Univariate analysis (HR (95% CI))	p-value	Multivariate analysis (HR (95% CI))	p-value
Age (years)	1.03 (0.98, 1.08)	0.25	1.04 (0.99, 1.09)	0.13	1.02 (0.97, 1.07)	0.31	1.03 (0.98, 1.08)	0.15
ALT (U/L)	1.01 (0.98, 1.03)	0.53	1.01 (0.99, 1.03)	0.4	1.00 (0.98, 1.02)	0.55	1.01 (0.98, 1.03)	0.42
Albumin (g/L)	0.89 (0.83, 1.04)	0.08	0.91 (0.85, 1.10)	0.17	0.92 (0.86, 1.08)	0.07	0.93 (0.88, 1.09)	0.16
Hypertension								
No	1	-	1	-	1	-	1	-
Yes	1.70 (0.85, 2.80)	0.21	1.60 (0.88, 2.65)	0.23	1.55 (0.80, 2.60)	0.21	1.50 (0.85, 2.50)	0.22
BMI (kg/m²)	0.96 (0.89, 1.05)	0.37	0.95 (0.88, 1.03)	0.27	0.94 (0.87, 1.04)	0.39	0.93 (0.87, 1.02)	0.28
Type 2 diabetes								
No	1	-	1	-	1	-	1	-
Yes	1.80 (0.85, 3.20)	0.11	1.75 (0.80, 3.10)	0.16	1.70 (0.78, 3.10)	0.12	1.60 (0.75, 3.00)	0.17
Liver cirrhosis								
No	1	-	1	-	1	-	1	-
Yes	1.35 (0.70, 2.20)	0.19	1.25 (0.65, 2.00)	0.22	1.30 (0.72, 2.15)	0.21	1.20 (0.65, 2.00)	0.24
Child-Pugh grade								
A	1	-	1	-	1	-	1	-
B	2.50 (1.20, 6.00)	0.03	2.30 (1.10, 5.60)	0.05	2.40 (1.15, 5.90)	0.04	2.15 (1.00, 5.60)	0.05
BCLC stage								
0/A	1	-	1	-	1	-	1	-
B	1.90 (0.70, 3.80)	0.15	1.75 (0.65, 3.60)	0.19	1.80 (0.65, 3.90)	0.16	1.60 (0.55, 3.60)	0.2
C	2.20 (1.15, 4.90)	0.08	2.00 (1.10, 4.50)	0.07	2.10 (1.10, 4.80)	0.09	1.90 (1.00, 4.50)	0.08
Tumour size (cm)	1.15 (1.05, 1.24)	0.02	1.12 (1.02, 1.22)	0.04	1.14 (1.05, 1.25)	0.02	1.10 (1.02, 1.20)	0.04
Tumour number								
Single	1	-	1	-	1	-	1	-
Multiple	2.00 (1.10, 3.80)	0.09	2.10 (1.20, 4.00)	0.06	2.10 (1.15, 3.90)	0.09	2.25 (1.15, 4.10)	0.07
Microvascular invasion								
No	1	-	1	-	1	-	1	-
Yes	2.20 (1.10, 3.90)	0.05	2.05 (1.05, 3.80)	0.06	2.30 (1.20, 4.10)	0.04	2.05 (1.10, 3.80)	0.06
Macrovascular invasion								
No	1	-	1	-	1	-	1	-
Yes	1.85 (1.10, 3.20)	0.08	1.75 (1.05, 3.10)	0.09	1.80 (1.10, 3.20)	0.08	1.75 (1.05, 3.	-

For the MAFLD group, BCLC stage B was significantly associated with recurrence in the multivariate analysis (HR = 1.80, p = 0.030). Tumour size was also significantly associated with recurrence in the univariate (HR = 1.10, p = 0.020) and multivariate (HR = 1.08, p = 0.080) analyses, with a trend towards significance in the latter. Microvascular invasion was a strong predictor in both the univariate (HR = 2.40, p = 0.010) and multivariate (HR = 2.30, p = 0.015) analyses. Macrovascular invasion showed a significant association with recurrence in the univariate analysis (HR = 2.10, p = 0.070), but not in the multivariate analysis.

In the non-MAFLD group, tumour size was a significant predictor of recurrence in both univariate (HR = 1.09, p = 0.010) and multivariate (HR = 1.10, p < 0.001) analyses. Microvascular invasion showed a strong association with recurrence in both univariate (HR = 2.25, p < 0.001) and multivariate (HR = 1.80, p < 0.001) analyses. Macrovascular invasion was also significantly associated with recurrence in the univariate analysis (HR = 2.15, p < 0.001), but not in the multivariate model. Additionally, the tumour number showed significance in the univariate (HR = 1.70, p = 0.010) and multivariate (HR = 1.50, p = 0.020) analyses (Table 3).

**Table 3:** Univariate and multivariable analyses of RFS in HCC patients LT, Alanine aminotransferase; BMI, body mass index; T2DM, type 2 diabetes mellitus; BCLC, Barcelona Clinic Liver Cancer; MAFLD, metabolic dysfunction-associated fatty liver disease, RFS, recurrence-free survival; HCC, hepatocellular carcinoma

	MAFLD group (n = 100)				Non-MAFLD group (n = 100)			
Variable	Univariate analysis (HR (95% CI))	p-value	Multivariate Analysis (HR (95% CI))	p-value	Univariate analysis (HR (95% CI))	p-value	Multivariate analysis (HR (95% CI))	p-value
Age (years)	1.00 (0.97, 1.03)	0.41	1.01 (0.98, 1.04)	0.34	0.99 (0.96, 1.02)	0.4	1.00 (0.98, 1.03)	0.35
ALT (U/L)	1.00 (0.99, 1.01)	0.33	1.00 (0.99, 1.01)	0.93	1.00 (0.99, 1.01)	0.35	1.00 (1.00, 1.01)	0.94
Albumin (g/L)	0.97 (0.91, 1.05)	0.46	0.98 (0.90, 1.06)	0.55	0.97 (0.92, 1.04)	0.45	0.98 (0.93, 1.05)	0.54
Hypertension								
No	1 (reference)	-	1 (reference)	-	1 (reference)	-	1 (reference)	-
Yes	1.10 (0.60, 2.00)	0.93	0.95 (0.70, 1.20)	0.42	1.05 (0.65, 1.85)	0.92	0.85 (0.60, 1.15)	0.37
BMI (kg/m²)	1.02 (0.94, 1.10)	0.46	1.00 (0.96, 1.07)	0.79	1.03 (0.95, 1.12)	0.46	1.00 (0.95, 1.05)	0.8
T2DM								
No	1	-	1	-	1	-	1	-
Yes	1.15 (0.55, 2.80)	0.68	1.05 (0.60, 2.25)	0.62	1.20 (0.52, 3.05)	0.69	0.95 (0.60, 1.45)	0.63
Liver cirrhosis								
No	1	-	1 (reference)	-	1	-	1	-
Yes	1.30 (0.75, 2.30)	0.37	1.10 (0.80, 1.60)	0.88	1.25 (0.70, 2.45)	0.37	0.95 (0.75, 1.25)	0.87
Child-Pugh grade								
A	1 (reference)	-	1 (reference)	-	1 (reference)	-	1 (reference)	-
B	1.20 (0.50, 3.15)	0.82	1.10 (0.55, 3.60)	0.94	1.65 (1.10, 2.55)	0.01	0.95 (0.60, 1.55)	0.78
BCLC stage								
0/A	1	-	1	-	1	-	1	-
B	1.95 (0.65, 9.00)	0.33	1.80 (1.00, 3.05)	0.03	2.10 (0.65, 9.20)	0.31	1.80 (1.05, 3.00)	0.03
C	1.85 (1.00, 3.55)	0.03	1.05 (0.80, 1.30)	0.9	1.90 (1.05, 3.65)	0.04	1.00 (0.75, 1.40)	0.89
Tumour size (cm)	1.10 (1.03, 1.17)	0.02	1.08 (0.99, 1.20)	0.08	1.09 (1.02, 1.18)	0.01	1.10 (1.05, 1.15)	<0.001
Tumour number								
Single	1	-	1	-	1	-	1	-
Multiple	1.65 (0.75, 3.50)	0.2	1.95 (0.80, 4.50)	0.14	1.70 (1.25, 2.30)	0.01	1.50 (1.10, 2.15)	0.02
Microvascular invasion								
No	1	-	1	-	1	-	1	-
Yes	2.40 (1.20, 4.30)	0.01	2.30 (1.10, 4.20)	0.015	2.25 (1.65, 3.05)	<0.001	1.80 (1.35, 2.30)	<0.001
Macrovascular invasion								
No	1	-	1	-	1	-	1	-
Yes	2.10 (0.90, 4.50)	0.07	1.20 (0.50, 3.00)	0.77	2.15 (1.50, 3.15)	<0.001	1.05 (0.70, 1.70)	0.78

**Figure 1:**
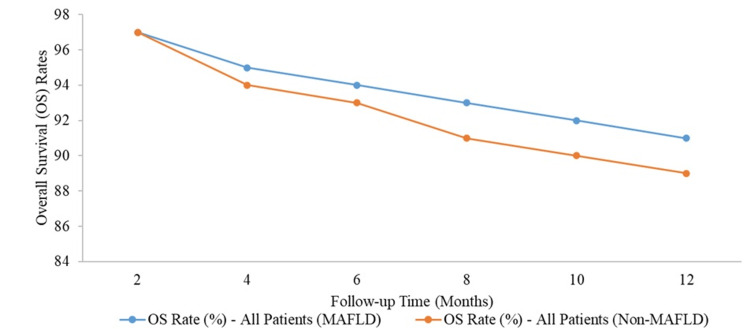
Kaplan-Meier analysis of overall survival for each counterpart Survival rate of all patients (MAFLD vs. non-MAFLD, p > 0.05). MAFLD, metabolic dysfunction-associated fatty liver disease

Both groups started with an OS rate of 97% at two months. However, as the follow-up progressed, the OS rate for non-MAFLD patients gradually declined more than for MAFLD patients. By the four-month mark, the OS rate for MAFLD patients was 95%, slightly higher than the 94% observed in non-MAFLD patients. This trend continued, with the most notable difference at 12 months, where MAFLD patients had an OS rate of 91%, compared to 89% in the non-MAFLD group (Figure 1).

**Figure 2:**
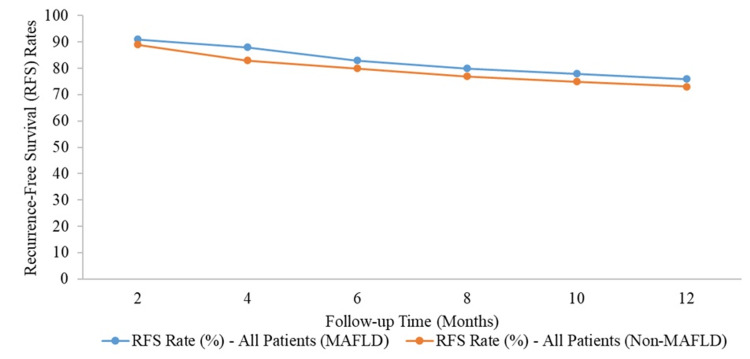
Kaplan-Meier analysis of recurrence-free survival for each counterpart Recurrence-free survival of all patients (MAFLD vs. Non-MAFLD, p > 0.05). MAFLD, metabolic dysfunction-associated fatty liver disease

Both groups had similar RFS rates initially, with MAFLD patients at 91% and non-MAFLD patients at 89% at the two-month mark. Over time, the RFS rates declined in both groups, but MAFLD patients consistently had slightly higher rates. At four months, MAFLD patients had an RFS rate of 88%, compared to 83% in non-MAFLD patients. By the end of the 12-month period, the RFS rate for MAFLD patients was 76%, whereas it was 73% for non-MAFLD patients. Despite these differences, the Kaplan-Meier analysis indicated that the RFS between the MAFLD and non-MAFLD groups was not statistically significant (p > 0.05) (Figure 2).

## Discussion

Our study investigated the impact of MAFLD on the prognosis of patients following radical resection of liver cancer in a well-matched cohort. Using a sizable database from the People's Hospital of Guangxi Zhuang Autonomous Region, we found that, while MAFLD had no discernible impact on RFS or male mortality, it significantly increased mortality in female HCC patients. Approximately 15% of the HCC cases in this study met the diagnostic requirements for MAFLD [20]. The significance of monitoring MAFLD-associated HCC among patients with cirrhosis is underscored by the fact that 64.6% of MAFLD-associated HCC patients did not have cirrhosis, suggesting that patients with MAFLD may develop HCC without cirrhosis [21,22].

In line with previous studies, our data showed that MAFLD-HCC cases had better OS and RFS compared to non-MAFLD-HCC cases, but this difference was not statistically significant (p > 0.05). For instance, the OS rate for MAFLD patients was 91% at 12 months, compared to 89% in non-MAFLD patients. Similarly, the RFS rate was 76% for MAFLD patients, versus 73% for non-MAFLD patients by the end of the 12-month period. These trends were consistent with a recent Italian study reanalysing 6,882 HCC cases using MAFLD criteria, which also found slightly better survival rates for MAFLD-HCC patients [23].

Our findings also showed significant differences in metabolic characteristics, with MAFLD patients having a higher BMI (25.3 kg/m² vs. 23.5 kg/m², p < 0.001) and a greater prevalence of type 2 diabetes (33% vs. 12%, p = 0.019) compared to non-MAFLD patients. ALT levels were elevated in the MAFLD group (median 38.0 IU/L vs. 32.0 IU/L, p = 0.045), indicating distinct metabolic profiles in MAFLD-HCC cases. Metabolic factors, like higher BMI and diabetes, are known to influence cancer prognosis, but recent research has shown they may not directly impact mortality in HCC cases [3,24,25]. Our analysis identified tumour size and microvascular invasion as significant predictors of HCC progression in both MAFLD and non-MAFLD groups. Notably, Child-Pugh grade B was a predictor of progression in both univariate and multivariate analyses for both groups.

A key gender-specific finding of this study was that metabolic dysfunction linked with MAFLD may have a more adverse effect on prognosis in women than in men. Women with MAFLD had higher mortality risk factors, including increased prevalence of diabetes and hypertension, similar to findings from global studies showing higher mortality from diabetes among women [26]. Consistent with studies from Japan and Canada, metabolic syndrome and MAFLD were linked to higher cancer-associated mortality in women [27]. Our findings also align with a recent Swiss cohort study that observed an increased prevalence of HCC in women with MAFLD [28]. The mechanism underlying the heightened cancer risk associated with MAFLD in women remains unclear but may involve obesity-related insulin resistance and insulin-like growth factor (IGF) pathways [29]. Additionally, the greater visceral adipose tissue (VAT) and increased free fatty acid release in women with MAFLD may contribute to higher cancer mortality risk [30,31].

Although our study provides valuable insights into gender-based differences in MAFLD-HCC prognosis, certain limitations persist. This single-institution, retrospective cohort study focused exclusively on surgical patients, which may limit the generalizability of our findings. Furthermore, our sample size - especially for women with MAFLD - was relatively small, potentially leading to selection bias. Future research with larger, more diverse samples and prospective designs is necessary to fully elucidate the relationship between MAFLD, gender, and HCC prognosis. Nonetheless, our study highlights that MAFLD significantly increases mortality risk for female HCC patients following radical resection, independently of RFS, and suggests a need for gender-specific monitoring and management in MAFLD-HCC cases.

## Conclusions

Our study demonstrates that, while MAFLD does not significantly impact OS or RFS following radical resection of HCC, it presents a distinct metabolic profile associated with increased BMI, prevalence of type 2 diabetes, and metabolic dysregulation. MAFLD appears to elevate mortality risk specifically among female HCC patients, likely due to gender differences in metabolic risk factors such as VAT and insulin resistance. These findings underscore the importance of tailored monitoring and management strategies in MAFLD-associated HCC, particularly for female patients. Further large-scale research is needed to clarify gender-based prognostic outcomes and improve survival rates.
